# Embryonic Protein Undernutrition by Albumen Removal Programs the Hepatic Amino Acid and Glucose Metabolism during the Perinatal Period in an Avian Model

**DOI:** 10.1371/journal.pone.0094902

**Published:** 2014-04-16

**Authors:** Els Willems, Tjing-Tjing Hu, Laura Soler Vasco, Johan Buyse, Eddy Decuypere, Lutgarde Arckens, Nadia Everaert

**Affiliations:** 1 Laboratory of Livestock Physiology, Department of Biosystems, KU Leuven, Leuven, Belgium; 2 Laboratory of Neuroplasticity and Neuroproteomics, KU Leuven, Leuven, Belgium; 3 Division of Livestock-Nutrition-Quality, Department of Biosystems, KU Leuven, Leuven, Belgium; 4 Animal Science Unit, Gembloux Agro-Bio Tech, University of Liège, Gembloux, Belgium; Wageningen UR Livestock Research, Netherlands

## Abstract

Different animal models have been used to study the effects of prenatal protein undernutrition and the mechanisms by which these occur. In mammals, the maternal diet is manipulated, exerting both direct nutritional and indirect hormonal effects. Chicken embryos develop independent from the hen in the egg. Therefore, in the chicken, the direct effects of protein deficiency by albumen removal early during incubation can be examined. Prenatal protein undernutrition was established in layer-type eggs by the partial replacement of albumen by saline at embryonic day 1 (albumen-deprived group), compared to a mock-treated sham and a non-treated control group. At hatch, survival of the albumen-deprived group was lower compared to the control and sham group due to increased early mortality by the manipulation. No treatment differences in yolk-free body weight or yolk weight could be detected. The water content of the yolk was reduced, whereas the water content of the carcass was increased in the albumen-deprived group, compared to the control group, indicating less uptake of nutrients from the yolk. At embryonic day 16, 20 and at hatch, plasma triiodothyronine (T_3_), corticosterone, lactate or glucose concentrations and hepatic glycogen content were not affected by treatment. At embryonic day 20, the plasma thyroxine (T_4_) concentrations of the albumen-deprived embryos was reduced compared to the control group, indicating a decreased metabolic rate. Screening for differential protein expression in the liver at hatch using two-dimensional difference gel electrophoresis revealed not only changed abundance of proteins important for amino acid metabolism, but also of enzymes related to energy and glucose metabolism. Interestingly, GLUT1, a glucose transporter, and PCK2 and FBP1, two out of three regulatory enzymes of the gluconeogenesis were dysregulated. No parallel differences in gene expressions causing the differences in protein abundance could be detected pointing to post-transcriptional or post-translational regulation of the observed differences.

## Introduction

Studies of the Dutch Hunger Winter (1944–1945) clearly showed that reduced growth *in utero* has detrimental effects on health in later life [1]. An association between a low birth weight and type II diabetes or impaired glucose tolerance has been found in people born around the time of the famine [2]. This means that poor nutrition *in utero* may lead to permanent changes in insulin-glucose metabolism. Indeed, by restricting the nutrient supply during the prenatal period, the fetus adapts to a low nutrient environment and makes metabolic adaptations to survive. However, when nutrition is adequate or overabundant in the postnatal life, a conflict between the programming and the postnatal conditions arises [3], [4]. The latter is referred to as the ‘fetal origins’ hypothesis' [5], which states that it is the conflict between the prenatal metabolic programming and the postnatal conditions that leads to disease and malfunction.

Prenatal protein undernutrition has been studied in several animal models. In these models, the maternal diet is manipulated, exerting direct nutritional effects as well as indirect effects such as hormonal changes on the fetus. As proposed by [6], the approach of albumen removal in avian eggs, as a model of prenatal protein undernutrition, offers a unique avian model to investigate the direct effect of reduced protein availability during embryogenesis on growth and metabolism. Recently, the effect of a low protein diet provided to the hens on metabolic programming of the offspring was investigated in the chicken [7]. Both the investigation of the direct (i.e. chicken embryos) and indirect animals models of prenatal protein deprivation can contribute to unraveling the prenatal programming effects. Several studies have already been conducted examining the effects of albumen removal in chicken for various reasons [6], [8], [9], [10]. A long-term study was previously conducted to examine the importance of albumen as a protein source during embryonic development in the chicken [9]. Before sexual maturation, the body weight and feed intake were reduced. In contrast, during adulthood, an increased body weight was accompanied by reduced reproduction performance (reduced laying rate and egg weight), indicating long-lasting programming effects.

The objective of the present study was to investigate if the chicken model of prenatal protein undernutrition already displays significant differences during the perinatal period, before the conflict between the prenatal and postnatal conditions arises and whether it is possible to detect effects on programming on the protein and gene expression during this period. For this purpose, 3 mL of albumen was removed from layer-type eggs and replaced with saline. During the perinatal period, the present model showed little differences in growth, hormones and metabolites and hepatic glycogen content, supporting the ‘fetal origins’ hypothesis'. However, metabolic programming caused by prenatal protein undernutrition was revealed by the observed hepatic proteome changes related with amino acids catabolism and glucose metabolism. Interestingly, the differential protein expression of these enzymes was not accompanied by a differential mRNA expression, suggesting that the observed proteome changes are related with post-transcriptional or post-translational events. The present results point to an interesting model, that can be used complementary to mammalian models for the further elucidation of the effects and mechanisms of prenatal protein undernutrition.

## Materials and Methods

### Ethics statement

All experiments were conducted in strict accordance with the European Communities Council Directive (2003/65/EC) and were approved by the Institutional Ethical Committee of KU Leuven (P132/2008).

### Experimental design

#### Incubation

A total of 528 fertilized Isa Brown layer-type eggs (Vepymo, Poppel, Belgium) from a 48-week-old breeder flock were individually numbered, weighed and randomLy divided over the three treatments. From 19 additional eggs, the albumen and yolk weight were determined. The eggs consisted of 26.6±0.4% yolk, 59.2±0.5% albumen and 14.2±0.3% egg shell. The eggs were incubated with the blunt end up in one forced-draft incubator (PAS Reform, Zeddam, the Netherlands) at a dry bulb temperature of 37.6°C and a wet bulb temperature of 29.0°C and were turned every hour over 90°. On embryonic day (**ED**) 18, all eggs were candled and those with living embryos were transferred from the turning trays to individual hatching baskets. After 22 days of standard incubation, the unhatched eggs were opened and checked for fertility or time of death in order to calculate survival percentage. Infertility was calculated as the number of infertile eggs relative to the total number of set eggs (%). Survival percentage was calculated as the number of hatched chicks relative to the number of fertile eggs (%). Early mortality was defined as dead embryos before ED 10 and mid mortality between ED 10–18. Embryos that died after ED 18 or were ready to hatch and alive in the shell, but had not hatched after 22 days of incubation, were classified as late embryonic mortality. Mortality rates were calculated relative to the number of fertile eggs (%).

#### Albumen removal

This method was previously described in [9]. Briefly, after one day of incubation (ED 1), albumen removal was established in 240 eggs (albumen-deprived group). After disinfection, a hole was drilled near the sharp end of the egg, 3 mL of albumen was replaced by the same volume of sterile saline, the hole was sealed using a drop of paraffin and the eggs were weighed. Albumen is the main source of protein for the developing embryo [11] and so the net effect is a prenatal protein undernutrition. A sham group of 144 eggs was mock-treated, similar to the albumen-deprived group, except for the actual albumen removal and saline injection. A third group of 144 eggs received no treatment (control group). The difference in the number of eggs for each treatment was based on the higher expected mortality due to albumen removal. To verify that the replacement of the albumen by saline was successful, the mean egg weight before incubation and after manipulation were compared.

### Sampling

At embryonic day (ED) 16 and 20 and at hatch, 15 embryos/chicks per group were weighed and killed by decapitation. The residual yolk was dissected and relative weights of both yolk and yolk-free body weight (YFBW) to egg weight at ED 1 were calculated. Weight of liver, heart, digestive tract and carcass was measured and proportional weights relative to YFBW were calculated. The digestive tract was defined as starting from the end of the esophagus, containing the proventriculus, the gizzard, the small and large intestine until the beginning of the cloaca. The carcass was defined as the YFBW minus liver, heart and digestive tract. Water content of yolk and carcass was determined by first drying at 65°C for 48 h and then at 105°C until a constant weight was reached.

At ED 16, blood was taken from the chorioallantoic membrane with a 1-mL syringe and 30 G needle and collected in tubes with heparine (Sigma, Bornem, Belgium). At ED20 and at hatch, blood was taken from the vena jugularis of the embryo or chick with a 1-mL syringe and 27 G needle and collected in tubes with heparine (Sigma, Bornem, Belgium). The blood was centrifuged at 1.500× g for 10 min at 6°C. The plasma was collected and stored at −20°C for later analyses of triiodothyronine (T_3_), thyroxine (T_4_), corticosterone, lactate and glucose. Liver samples were taken and snap frozen in liquid nitrogen before storage at −80°C. Frozen liver tissue was homogenized by grinding into powder in liquid nitrogen before use.

### Hormones and Metabolites

Plasma analysis was performed on 15 samples per group per sampling day for each measurement. Plasma T_3_ and T_4_ concentrations were measured by radio-immunoassay (RIA) as described by [12]. The antisera for T_3_ and T_4_ were purchased from Byk-Belga (Brussels, Belgium). Intra-assay coefficients of variation are 4.5% and 5.4% for T_3_ and T_4_ respectively. Corticosterone was measured with the Corticosterone Double Antibody – 125I RIA Kit for Animal Testing (07120103, MP Biomedicals, Carlsbad, California, USA). The intra-assay coefficient for corticosterone is 7.1%.

Plasma lactate was measured using a commercially available kit (LAT8840, Ben S.r.l.-Biochemical Enterprise, Milan, Italy) following the manufacturer's instruction. The intra-assay coefficient for lactate is 2.3%. Plasma glucose concentration was determined using a commercially available kit (298–65701, WAKO Pure Chemical Industries Ltd., Osaka, Japan). The absorbance was measured at 490 nm (Victor 1420 Multilabel counter, PerkinElmer, MA, USA). The intra-assay coefficient for glucose is 2.2%.

### Glycogen determination

For the determination of the hepatic glycogen content, a method adapted from [13] was used. Tissue was homogenized in exactly the same volume (µL) 7% HClO_4_ as mg tissue was taken. Homogenates were centrifuged at 4°C at 14,000 g until a clear supernatant was obtained. The supernatant was washed with 1 mL of petroleum ether and stored at −80°C. An iodine color reagent [0.39 mL of an iodine solution (0.104 g I_2_, 1.04 g KI_2_ in 4 mL of milli-Q water) +30 mL of 10% CaCl_2_] was added to standards or tissue extracts in a microtiter plate. After mixing and an incubation period of 10 min, the absorbance was measured at 450 nm (Victor 1420 Multilabel counter, PerkinElmer, MA, USA). Tissue glycogen concentration (n = 12) could then be calculated using a standard curve of rabbit liver glycogen (Sigma, Bornem, Belgium). Total glycogen content (ng) was calculated by multiplying the glycogen concentration (ng/mg) with the wet weight of the liver (mg).

### Two-dimensional difference gel electrophoresis (2-D DIGE)

The 2-D DIGE was performed as previously described [14]. The fluorescent cyanine dyes (Cy2, propyl-Cy3 and methyl-Cy5) were synthesized as described previously [15], [16] according to the method by [17]. All other chemicals were purchased from GE Healthcare, unless mentioned otherwise.

#### Protein lysates

100 mg liver tissue taken at hatch (n = 6 per group) was transferred to 500 µL ice-cold lysis buffer, containing 7 M urea, 2 M thiourea, 4% w/v CHAPS (Sigma-Aldrich), 1% w/v DTT (Serva), 40 mM Tris base (ICN; Aurora), pH 8 and Complete Protease Inhibitor Cocktail (Roche Diagnostics, Basel, Switserland). Liver tissue was homogenized on ice, briefly centrifuged at 13,000 rpm, sonicated (3 times 25 sec), followed by a complete solubilization of the proteins for 1 h at room temperature. The proteins were sonicated again briefly and centrifuged for 20 min at 13,000 rpm at 4°C to precipitate cell debris. The supernatant was dialyzed against milli-Q water for 2 h to remove residual salt using a membrane with a 500-Da cutoff (Spectra/Por, Biotech, Omnilabo) and aliquots were stored at −80°C. Protein concentrations were determined with the Quant-iT Protein Assay kit (Q33211, Invitrogen, Carlsbad, CA, USA) using the Qubit Fluorometer 2.0 (Q32866, Invitrogen).

#### Analytical gels

Pre-cast Immobiline DryStrips (24 cm, pH 3–11 nonlinear) were rehydrated overnight in DeStreak Rehydration Solution containing 0.5% v/v immobilized pH gradient (IPG) buffer in a reswelling tray covered with paraffin oil (Merck). The next day 50 µg protein of each liver samples (n = 6 per group) was randomLy labeled with either propyl-Cy3 (n = 3) or methyl-Cy5 (n = 3) dissolved in dimethylformamide (DMF). The samples were incubated for 30 min on ice in the dark to achieve minimal labeling of proteins with approximately 200 pmol dye and the highest signal-to-noise ratio and maximal number of labeled spots. Labelling was terminated by addition of 1 µL lysine (10 mM; Merck) for 15 min on ice in the dark. Equal fractions of all 18 samples were pooled and per gel 50 µg of this pool was labeled with 200 pmol Cy2 dissolved in DMF to serve as an internal standard. In total, 9 gels were run. The Cy2-, Cy3-, and Cy5-labeled fractions were mixed together, and an equal volume of lysis solution was added. Isoelectric focusing (IEF) was performed in 24-cm long pre-cast Immobiline DryStrips over a pH range of 3–11 (non-linear) on an Ettan IPGphor Cup Loading Manifold system according to manufacturer's instructions. Actual run conditions were 300 V for 3 h, 600 V for 3 h, followed by a 6-h gradient to 1000 V, a 3-h gradient to 8000 V, and 8 h at 8000 V for a total of 75–85 kVh (at 50 µA/strip). After IEF, the strips were reduced with DTT (1% w/v) in equilibration buffer (6 M urea, 34.5% v/v glycerol and 10% w/v SDS in Tris-HCl buffer [1.5 M, pH 8.8]) for 15 min followed by alkylation with 4.5% w/v iodoacetamide (Sigma-Aldrich) in equilibration buffer for 15 min. Electrophoresis of the IPG strips was done on 1.5-mm-thick SDS-polyacrylamide gels (12.5% T; 2.6% C) in the Ettan DALT twelve system for 30 min at 30 mA and 24 h at 15 mA/gel at 13°C. The 2-D DIGE gel plates were rinsed with milli-Q water.

#### Image analysis

Gels were scanned with the Ettan DIGE Imager (software 1.0; GE Healthcare) and generated gel image triplets (Cy2, Cy3, and Cy5) comprising the CyDye-labeled proteins. Quantitative analysis was carried out with the DeCyder 2D difference analysis software (Version 6.5; GE Healthcare). Spot detection and matching was performed automatically with the DeCyder Batch processor. For spot detection, the estimated number of spots for each co-detection procedure was set to 2500. The best internal standard image (Cy2 labeled samples) based on the number of detected spots and overall similarity of the protein spot pattern with that of other gels was assigned as the “Master” and used as a template. The matching was checked manually in the biological variation analysis (BVA) module to ascertain the accuracy of the match process. Volume ratios were calculated for every spot on every gel by dividing the spot volume (Cy3 or Cy5), by the spot volume of the internal standard (Cy2), thereby correcting for inter-gel variations.

#### Preparative gels and protein identification

Two preparative gels were run under the same conditions as described above. Each gel was loaded with 1.5 mg of protein from the pool sample, from which only a 50 µg fraction was labeled with Cy3. Glass plates were pretreated with BindSilane, and 2 reference markers were applied to enable automatic spot picking. The preparative gels were scanned in the Ettan DIGE Imager to obtain an image of the Cy3 signal. Subsequently, the total protein load was visualized by fluorescent LavaPurple Total protein stain according to the manufacturer's instructions (GelCompany, San Fransisco, California, USA), and the gels were scanned again. Matching with the analytical gels was carried out automatically with manual correction by the BVA module of the DeCyder software. A pick list of the proteins of interest was generated and imported into the Spot Picker Version 1.20 software that controls the Ettan Spot Picker (GE Healthcare, Little Chalfont, UK) and the relevant spots were excised. All subsequent steps were carried out under a laminar flow hood in dust-free conditions to prevent keratin contamination of the samples. Identical spots from the two preparative gels were pooled. Before tryptic digestion, the gel pieces were thoroughly rinsed with milli-Q water, 50% v/v acetonitrile (ACN) (Biosolve) and again with milli-Q water. After drying in a SpeedVac vacuum centrifuge, they were incubated overnight at 37°C in a 40 µL digestion solution containing 100 ng modified porcine trypsin (sequencing grade, Promega, Fitchburg, Wisconsin, USA), 25 mM NH_4_HCO_3_, and 5% v/v ACN. Tryptic peptides were extracted from the gel pieces by 2 washing steps of 30 min in a bath sonicator. In the first step, a 100 µL solution of 5% v/v ACN and 0.5% formic acid (FA) (Riedel-de-Haën), and the second step was carried out in a 50 µL solution containing 10% ACN and 0.5% FA. The supernatant was concentrated to a volume of approximately 20 µL using SpeedVac vacuum centrifugation. Automated LC-MS analyses were run on an Ultimate 3000 Nano LC System (Dionex) coupled on-line to a microTOF-Q mass spectrometer (Bruker Daltonics). The total volume of each sample was pipetted in a polypropylene vial (Dionex, LC-Packings) and loaded in the LC autosampler. 5 µL of this peptide solution was transferred to the precolumn (C18 PepMap100 internal diameter (i.d.) 300 lm 3 5 mm; Dionex, LC Packings) and reversed phase eluted over a 75 µm i.d.×15 cm PepMap 100 C18 nanocolumn at a flow rate of 200 nL/min, using a linear gradient from 5% to 40% of ACN/0.5% FA in 25 min. The column outlet was coupled to the Q-TOF through a stainless steel needle at 2000 V. The ionized peptides in the range of 400–1400 m/z were automatically selected and fragmented using predefined collision energy profiles, depending on the detected peptide charge. LC-MS data were processed using the Data Analysis software version 3.4 of Bruker Daltonics and submitted to the Mascot MS/MS ions search engine using the same parameters. For protein identification, the SwissProt 51.6 database (limited to Chordata) was used and a 95% confidence interval threshold (P<0.05) was set. The molecular function and subcellular location of these proteins were examined using UNIPROT.

#### Identification of relevant canonical pathways

The list of differentially expressed proteins was imported into the Ingenuity Pathway Analysis (IPA; Ingenuity Systems, Redwood City, CA, USA) to identify biological interactions between the proteins. The biological interaction scores were defined by the IPA statistical algorithm based on its knowledge base, and the P-value was corrected for multiple comparisons with the Benjamini–Hochberg test.

### Quantitative real-time PCR

#### RNA extraction and reverse transcription

Total RNA was extracted using 750 µL of TRIzol (Invitrogen, Paisley, UK) added to approximately 50 mg of crushed liver sample taken at hatch (n = 8 per group), and incubated for 5 min at room temperature. 300 µL of chloroform (Sigma Aldrich, Missouri, USA) was added to create a phase separation. After a short vortex step, the samples were centrifuged at 14,000 rpm for 15 min at 4°C. The aqueous phase was transferred to fresh tubes, mixed with 375 µL ice-cold isopropanol (Prolabo, VWR, West Chester, Pennsylvania, USA) and incubated for 10 min at room temperature. After centrifugation (14,000 rpm, 10 min, 4°C), the supernatant was discarded and the remaining RNA pellet was washed with 750 µL ice-cold 70% ethanol (Prolabo, VWR, West Chester, Pennsylvania, USA). After a final centrifugation step (7,500 rpm, 5 min, 4°C), the supernatant was carefully removed and the remaining RNA pellet was dissolved in 1∶1000 DEPC-treated water (Fluka). The RNA concentration and quality (260/280 ratio) was measured using UV-spectroscopy (Implen, Westburg, Leusden, The Netherlands). The integrity of the RNA was electrophoretically verified using 2% agarose (Sigma Aldrich, Missouri, USA) gel electrophoresis and Midori Green DNA Stain (MG02, NIPPON Genetics Europe GmbH, Dueren, DE).

The RNA was transcribed into cDNA using the Reverse Transcription system (A3500, Promega, Madison, WI, USA). Denaturation was performed for 3 min at 80°C followed by 45 min at 42°C for the reverse transcription. The reaction was stopped by heating the samples for 5 min at 95°C.

#### Primer design

The intron-spanning primers for the different genes were designed using Primer Designing Tool (NCBI). The primers were purchased from IDT (Integrated DNA Technologies Inc., Coralville, Iowa, USA). In table 1, all primers are listed, both for reference genes and the genes of interest. For every pair of primers used, standard curves were made and PCR efficiencies were calculated according to the following formula: PCR efficiency = −1+10^−1/slope^ [18]. A PCR efficiency between 90% and 110% is generally considered acceptable, with correlation coefficient >0.99. Final primer concentration was 0.3 µM for all primer pairs.

**Table 1:** List of primers used for quantification of the genes of interest and reference genes.

Gene	Accession number		5′-primer-3′	Tm (°C)	Size (bp)	Efficiency (%)
PCK2	NM_205470	F	GGCCGAGCACATGCTGATTT	61.7	62	102.7
		R	CCGCCATGTAACGCTTCTCA	60.7		
PCK1	NM_205471	F	GGAAGTAGCCAGCTGGGTCGC	59.5	91	105.1
		R	TTGTGCGTCCTTGCATGCAGC	59.1		
FBP1	NM_001278048	F	TGCTGCGGTCACCGAGTATCTCA	59.8	56	95.6
		R	GCGAACTGCCGTCCTCAGGG	59.8		
HIBADH	NM_001006362	F	CCTGGGTGCTCAGGTAACAG	60.0	84	99.4
		R	TTGGGACTTGAAGGCAGCAT	59.9		
ACTB	NM_205518	F	TCGCCCCAGACATCAGGGTGTGA	61.5	75	103.3
		R	TTGCTCTGGGCTTCATCACCAACGT	60.9		
PPID	XM_426283	F	GTCGCACCCGTCCCCTGTAGA	60.0	93	101.8
		R	ATTCGTCCAACTCGCTCTCCCC	58.4		

For every gene the NCBI accession number, the sequences and the theoretical melting temperature (Tm, °C) of the forward (F) and reverse primer (R), the size of the amplified fragment (bp) and the efficiency of amplification (%) are provided.

#### qRT-PCR

Quantitative real-time PCR (qRT-PCR) measurements were performed in duplicate using the ABI StepOnePlus (Applied Biosystems, Carlsbad, USA) and Maxima SYBR Green/ROX qPCR Master Mix (2x, Fermentas Life Sciences, St. Leon-Rot, Germany). 1 µL of 1∶2 diluted cDNA was used as input in 25 µL reaction. The PCR reaction program began with 10 min heating at 95°C followed by 40 cycles of 15 seconds at 95°C and 60 seconds at 60°C. For the analysis of the qRT-PCR output, the 2^−ΔΔCT^ method of relative quantification was used [19]. Expression of genes was normalized to the geometric average of the two references genes: β-actine (ACTB) and peptidylprolylisomerase D (PPID). In addition, a melting curve analysis was performed to check the specificity of the primers (15 seconds at 95°C, 1 minute at 60°C, temperature gradually increased by 0.3°C until 95°C is reached).

### Statistical Analysis

All data were processed with the statistical software package SAS version 9.2 (SAS Institute Inc., Cary, NC). A GLM was used to analyse the effect of treatment (control, sham and albumen-deprived group), age (ED 16, ED 20 and at hatch) and their interaction on the measured parameters: absolute and relative YFBW and residual yolk weight, water content of the residual yolk and carcass, absolute and proportional liver, digestive tract and heart weight, plasma T_3_, T_4_, corticosterone, glucose and lactate concentrations. The liver glycogen content, logarithms of the volume ratios (Cy3/Cy2 or Cy5/Cy2) of the protein spots of 2-D DIGE and the fold change of phosphoenolpyruvate carboxykinase 1 (PCK1) and 2 (PCK2), fructose-1,6-bisphosphatase 1 (FBP1) and 3-hydroxyisobutyrate dehydrogenase (HIBADH) were analysed using the one-way ANOVA with treatment as variable. When there was a significant overall effect of treatment, or interaction with age, the means were further compared by a post-hoc Tukey's test. Survival rate, early, mid and late embryonic mortality, and infertility were analyzed using the logistic regression model with treatment as the classification variable. For all parameters, a degree of significance of 5% was used. All data are shown as mean ± SEM.

## Results

### Survival and mortality

Survival until hatch (Table 2) differed between all three experimental groups. The albumen-deprived group had a lower survival compared to both the control (P<0.001)) and the sham group (P<0.001). Furthermore, the survival was also reduced in the sham group compared to the control group (P = 0.001). The albumen-deprived group had a higher early mortality (Table 2) than the control group (P<0.001), with the sham group being intermediate and different from both the control (P<0.001) and the albumen-deprived group (P<0.001). No differences between treatments were found for the mid or late mortality (Table 2). Infertility was similar in all treatments (Table 2).

**Table 2:** Survival percentage (%), early, mid and late mortality (%) and infertility (%) of the eggs of the control, sham and albumen-deprived group.

Item (%)	control	sham	albumen-deprived	P-value
Survival	87.7^a^	69.3^b^	38.1^c^	<0.001
Early mortality	7.0^a^	25.4^b^	55.7^c^	<0.001
Mid mortality	0.0	0.0	0.5	NS
Late mortality	5.3	5.3	5.7	NS
Infertility	2.6	4.2	2.3	NS

Number of fertile eggs in control (n = 144), sham (n = 144) and albumen-deprived group (n = 240).

a–c Within a row, treatment means with different superscript are significantly different (P<0.001). P-values of effect of treatment are added in a separate column.

Survival (%) =  (number of hatched chicks/number of fertile eggs)*100

Mortality (%)  =  (number of dead eggs/number of fertile eggs)*100

Infertility (%)  =  (number of infertile eggs/total number of set eggs) *100

### Yolk-free body and residual yolk weight

The absolute and relative (to egg weight at ED1) YFBW and residual yolk weight (Table 3) were only affected by age (P<0.001) but not by treatment or the interaction. The water content of the yolk (Table 3) however, was affected by treatment (P = 0.016), but not by age or the interaction. The albumen-deprived group had a lower water content of the yolk compared with the control group (P = 0.012), whereas the sham group was not different from either the control or the albumen-deprived group.

**Table 3:** Yolk-free body weight (YFWB) (g), relative YFBW to egg weight at embryonic day 1 (ED1) (%), absolute (g) and relative residual yolk to egg weight at ED1 (%) at ED 16, 20 and at hatch of the control, sham, and albumen-deprived group (n = 15 per group per age).

	control		sham		albumen-deprived		P-value treatment	P-value age	P-value interaction
	Mean	SEM	Mean	SEM	Mean	SEM			
YFBW weight (g)	28.7	1.3	29.0	1.3	28.5	1.4	NS	<0.001	NS
Relative YFBW weight (%)	48.3	2.2	47.6	2.2	47.0	2.2	NS	<0.001	NS
Residual yolk weight (g)	9.0	0.5	9.1	0.5	8.8	0.5	NS	<0.001	NS
Relative residual yolk weight (%)	14.9	0.7	14.7	0.7	14.8	0.8	NS	<0.001	NS
Water content residual yolk (%)	52.4^a^	0.5	51.8^ab^	0.4	50.8^b^	0.4	0.016	NS	NS

a–b Within a row, treatment means with different superscript are significantly different (P<0.05). P-values of effect of treatment, age and interaction are added in separate columns. Data are shown as mean ± SEM.

### Body composition

Only age had an effect on the absolute liver, absolute carcass, absolute and proportional digestive tract and absolute and proportional heart weight (data not shown). The proportional liver (to YFBW) weight, however, was affected by age (P<0.001) and almost by treatment (P = 0.057), but not by the interaction. The albumen-deprived chicks had a numerically lower proportional liver weight (2.00±0.03%) than the control (2.10±0.04%) and the sham group (2.08±0.04%). The proportional carcass weight was affected by treatment (P = 0.024), age (P<0.001) and the interaction (P = 0.026). At hatch, the albumen-deprived chicks had a higher proportional carcass weight (84.0±0.3 g) than the control (82.1±0.4 g, P = 0.032) and the sham group (82.1±0.4 g, P = 0.034). The water content of the carcass was affected both by age (P<0.001) and treatment (P = 0.017). The albumen-deprived chicks had a higher water content of the carcass (82.2±0.3%) than the control chicks (81.3±0.3%, P = 0.014), whereas the sham group (81.6±0.3%) was not different from both other groups.

### Plasma hormones and metabolites and liver glycogen

**Figure 1:**
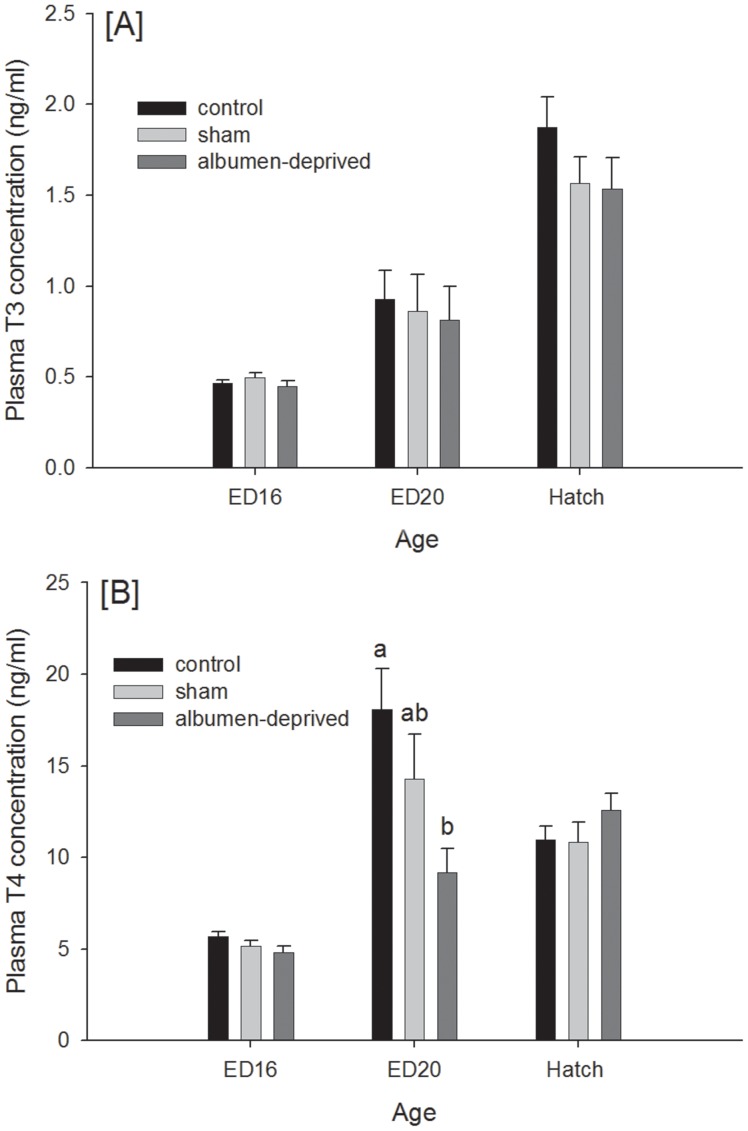
Plasma thyroid hormones. T_3_ (A) and T_4_ (B) concentrations (ng/mL) of the control, sham and the albumen-deprived chicks at embryonic day (ED) 16, ED 20 and at hatch (n = 15). Data are shown as mean ± SEM.^a–b^ Treatment means differ per timepoint (P<0.05).

**Figure 2:**
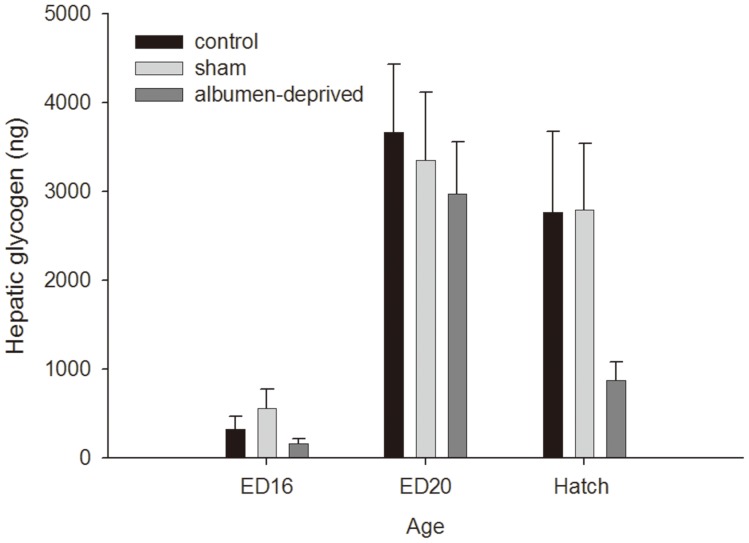
Glycogen in the liver. Hepatic glycogen content (ng) of the control, sham and the albumen-deprived chicks at embryonic day (ED) 16, ED 20 and at hatch (n = 12). Data are shown as mean ± SEM.

Only an effect of age (P<0.001) was observed on the plasma T_3_ concentrations, with concentrations increasing with age (Figure 1A). The plasma T_4_ levels (Figure 1B) were affected by treatment (P = 0.033), age (P<0.001) and the interaction (P = 0.001). On ED20, the albumen-deprived group had lower T_4_ levels than the control group (P<0.001). Corticosterone levels were not influenced by age, treatment or the interaction (data not shown). Glucose and lactate levels were only affected by age (P<0.001, data not shown). The glycogen content of the liver (Figure 2) had an effect of age (P<0.001) but not of treatment or the interaction.

### Screening for differential proteins in liver samples at hatch using 2-D DIGE

**Figure 3:**
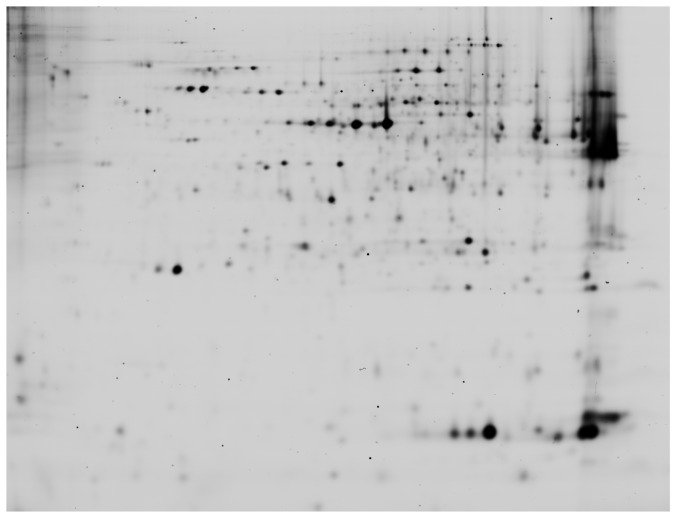
Hepatic proteome of the newly-hatched chick. Visualization of the total number of spots in the hepatic proteome of the newly-hatched chick on a Cy2 image (pooled sample) of an analytic two-dimensional gel electrophoresis (2-D DIGE) gel.

Of the approximately 1400 spots present on the gels (Figure 3), 15 spots were affected by treatment (P<0.05). Spots exhibiting differences between the control and the sham group were excluded, as these differences are induced by the manipulation of the egg and not the prenatal undernutrition effect. Only the spots for which the expression in the albumen-deprived group differed from the control group (4 spots), the sham group (1 spot) or both (3 spots) were selected for identification by mass spectrometry. Table 4 shows the proteins that were identified in each of the spots. Four of these protein spots were upregulated, while 4 were downregulated in the liver of albumen-deprived chicks. Key metabolic pathways and protein networks affected by the treatment of prenatal protein undernutrion by albumen removal in the chicken were identified by systems biology analysis using Ingenuity Pathways analysis (IPA) software. Relevant pathways are listed in Table 5. Several of these pathways are involved in the degradation of amino acids such as valine, methionine, cysteine and glutamate. Moreover, pathways direct or indirect involved in the glucose metabolism are also apparent: glycolysis/gluconeogenesis, tricarboxylic acid cycle (TCA) and fermentation of pyruvate to lactate. Finally, activation of TR/RXR (thyroid and retinoid X receptor) and FXR/RXR (farnesoid X receptor, retinoid X receptor, cholesterol homeostasis) were detected.

**Table 4:** Identification with LC-MS/MS of the proteins present in the 4 upregulated and 4 downregulated spots in the liver of albumen-deprived chicks at hatch compared to either the control chicks or the sham chicks or both (n = 6).

Gene	Full name	Spot	Uniprot ID	pI	Mass(kDa)	Mascot Score	Queries	Coverage(%)	Location	control	sham	albumen-deprived	P-value
**UP**													
HSPA9	Stress-70 protein, mitochondrial precursor	407	Q5ZM98	6.09	73,2	188	5	10	Mt	1.00±0.09^b^	1.10±0.07^ab^	1.44±0.12^a^	0.010
ALB	Serum albumin		P19121	5.51	69,9	145	16	26	Pl				
HSPA2	Heat shock 70 kDa protein		P08106	5.52	69,8	137	4	9	Cs				
ALB	Serum albumin	439	P19121	5.51	69,9	88	12		Pl	1.00±0.11^b^	1.04±0.07^ab^	1.38±0.14^a^	0.035
PCK2	Phosphoenolpyruvate carboxykinase2	232	P21642	7.56	71,1	364	10	16	Mt	1.00±0.05^b^	1.12±0.04^ab^	1.28±0.09^a^	0.021
LMNA	Lamin-A		P13648	6.50	73,2	113	2	3	Mt, Nu				
ENO1	Alpha-enolase§		P51913	6.17	47,3	112	3	10	Cp				
TUFM	Elongation factor Tu		P84172	8.98	38,3	52	1	3	Mt				
MDH1	Malate dehydrogenase 1		Q5ZME2	6.92	36,5	36	1	3	Cp				
ACO2	Aconitate hydratase§	274	Q8AYI3	8.04	85,8	261	6	9	Mt	1.00±0.03^b^	1.01±0.02^b^	1.12±0.04^a^	0.010
DLD	Dihydrolipoyl dehydrogenase§		Q5ZM32	8.19	54.0	138	6	14	Mt				
GLUD1	Glutamate dehydrogenase 1		P00368	8.48	55,7	65	4	12	Mt				
ALB	Serum albumin§		P19121	5.51	69,9	69	1	3	Pl				
PCK2	Phosphoenolpyruvate carboxykinase		P21642	7.56	71,1	59	1	2	Mt				
CTH	Cystathionine gamma-lyase§		E1BYF1	6.85	43,9	51	1	3	Cp				
GLUT1	Solute carrier family 2, facilitated glucose transporter member 1		P46896	8.82	54,1	42	2	1	Cm				
SMAD3	Mothers against decapentaplegic homolog 3		P84023	6.70	48,3	37	1	3	Cp, Nu				
LDHB	L-lactate dehydrogenase B chain		P00337	7.07	36,3	36	1	3	Cp				
**DOWN**													
RPLP0	60S acidic ribosomal protein P0§	1033	P47826	5.71	34,3	42	1	3	Rb	1.00±0.07^ab^	1.04±0.09^a^	0.78±0.07^b^	0.050
FBP1	Fructose-1,6-bisphosphatase 1§	1045	Q9I8D4	5.22	18,2	81	3	7	Cp	1.00±0.10^a^	0.89±0.08^ab^	0.63±0.12^b^	0.036
HIBADH	3-hydroxyisobutyrate dehydrogenase §	1126	Q5ZLI9	8.60	35,3	421	10	16	Mt	1.00±0.10^a^	0.99±0.06^a^	0.58±0.14^b^	0.011
TST	Thiosulfate sulfurtransferase	1135	P25324	6.56	32,3	92	7	15	Mt	1.00±0.20^a^	1.16±0.16^a^	0.42±0.16^b^	0.007

The spot number (Spot), Uniprot accession number (Uniprot ID), theoretical iso-electric point (pI), theoretical molecular weight (Mass), Mascot score, number of matched peptides (Queries) and sequence coverage (Coverage) are shown. The cellular localization (Location) is also indicated: Cm  =  cell membrane, Cp  =  cytoplasm, Cs  =  cell surface, Mt  =  mitochondria, Nu  =  nucleus, Pl  =  plasma and Rb  =  Ribosome. The mean fluorescence (Cy3 or Cy5) of the different spots normalized according to the fluorescence signal of the pooled sample (Cy2) ± SEM are shown in the table for the control, sham and albumen-deprived chicks. ^a–b^ Within a row, treatment means with different superscript are significantly different (P<0.05). P-values of effect of treatment are added in a separate column. Most of the identified spots contained just one protein, but several enclosed multiple proteins. On the other hand, some proteins were present in more than one spot. Porcine trypsine was added for fragmentation before LC-MS/MS and this was consequently identified in the samples but this was omitted for clarity. §Homologous protein was identified in different species, but results are always displayed for the chicken (*Gallus gallus*).

**Table 5:** Identification of relevant canonical pathways affected by albumen removal by grouping of the differential expressed proteins through the use of Ingenuity Pathway Analysis (IPA).

	Molecules	B-H P-value	Ratio
Ingenuity canonical pathways			
TCA cycle II	ACO2, DLD, MDH1	3.55E-04	3/41
Gluconeogenesis I	ENO1, FBP1, MDH1	3.55E-04	3/48
Glucocorticoid receptor signaling	PCK2, SMAD3, HSPA9, HSPA2	2.57E-03	4/299
Valine Degradation I	HIBADH, DLD	8.13E-03	2/35
Glycolysis I	ENO1, FBP1	8.51E-03	2/41
Superpathway of Methionine Degradation	DLD, CTH	1.82E-02	2/64
TR/RXR Activation	ENO1, GLUT1	2.88E-02	2/109
FXR/RXR Activation	PCK2, FBP1	2.88E-02	2/110
L-cysteine degradation II	CTH	2.88E-02	1/5
Glutamate Biosynthesis/Degradation X	GLUD1	2.95E-02	1/7
Pyruvate Fermentation to Lactate	LDHB	2.95E-02	1/9

IPA-analysis (www.ingenuity.com) was used to identify key biological pathways comprising the differentially identified proteins after prenatal protein undernutrition by albumen removal in chicken. The significance of the canonical pathways was tested by the stringent Benjamini-Hochberg (B-H) multiple testing correction method. The ratio indicates the number of differential proteins in a given pathway divided by the total number of molecules that make up that pathway. The following proteins, annotated with their gene names, are included in the canonical pathways. Abbreviations: ACO2 (Aconitate hydratase); CTH (Cystathionine gamma-lyase); DLD (Dihydrolipoyl dehydrogenase); ENO1 (Alpha-enolase); FBP1 (Fructose-1,6-bisphosphatase 1); GLUD1 (Glutamate dehydrogenase 1); GLUT1 (Solute carrier family 2, facilitated glucose transporter member 1); HIBADH (3-hydroxyisobutyrate dehydrogenase); HSPA2 (Heat shock 70 kDa protein); HSPA9 (Stress-70 protein, mitochondrial precursor); LDHB (L-lactate dehydrogenase B chain); MDH1 (Malate dehydrogenase); PCK2 (Phosphoenolpyruvate carboxykinase); SMAD3 (Mothers against decapentaplegic homolog 3).

### Gene expression of PCK1, PCK2, FBP1 and HIBADH

To investigate if differences in gene expression might cause the difference in protein abundance, several proteins were selected and the mRNA expression was examined. No effect of treatment could be detected on the gene expression of PCK1, PCK2, HIBADH or FBP1 (Table 6), although a trend was observed towards decreased expression of FBP1 (P = 0.094). The expression of FBP1 in the liver of albumen-deprived chicks at hatch tended to be reduced compared to the control or sham chicks.

**Table 6:** Gene expression of PCK1, PCK2, HIBADH and FBP1 normalized to the geometric average of expression of ACTB and PPID of the control, sham and the albumen-deprived chicks (n = 8) in the liver of newly-hatched chicks.

	control		sham		albumen-deprived		P-valuetreatment
	Mean	SEM	Mean	SEM	Mean	SEM	
HIBADH	1.00	0.10	0.95	0.06	0.87	0.05	NS
PCK1	1.00	0.50	0.79	0.26	1.08	0.44	NS
PCK2	1.00	0.05	0.96	0.08	0.94	0.07	NS
FBP1	1.00	0.04	0.97	0.06	0.85	0.04	0.094

Data are shown as mean ± SEM.

## Discussion

Our previous study demonstrated the existence of long-term effects of prenatal protein undernutrition by albumen removal on the body weight and reproduction performance of laying hens [9]. According to the ‘fetal origins’ hypothesis' [5], it is the conflict between prenatal environment (i.e. reduced protein availability) and the postnatal conditions (i.e. adequate protein levels) that causes disease and malfunction in later life. Indeed, by restricting the nutrient supply during the prenatal period, the fetus adapts to a low nutrient environment and makes metabolic adaptations to survive. However, when nutrition is adequate or overabundant in the postnatal life, a conflict between the programming and the postnatal conditions arises. The objective of the present study was to investigate if the chicken model of prenatal protein undernutrition by albumen removal already displays metabolic programming effects during the late embryonic period until hatch (before access to feed), before the conflict is created.

Effects on YFBW and body composition were examined as phenotypic parameters. In the current study, the equal absolute and relative YFBW and yolk weight together with a lower water content of the yolk (and hence higher weight of dry matter in the yolk) of the albumen-deprived chicks, would suggest a lower utilisation of solids to obtain an equal body weight. In contrast, results from [9] implied a higher consumption of the available yolk by the albumen-deprived group to support an equal embryonic growth until hatch. This discrepancy is remarkable, however, the higher water content of the carcass (and hence less solids in the carcass) is in agreement with increased amount of solids remaining in the yolk, as less were used to synthesize tissue. In addition, no differences in body composition was observed in the albumen-deprived chicks except for a marginally lower proportional liver weight together with an equal YFBW and reduced carcass weight. These results are in agreement with the ‘fetal origins’ hypothesis' [5]. Since these investigations are focused on the perinatal period, no conflict between the prenatal prenatal (i.e. reduced protein availability) and postnatal environment (i.e. adequate protein levels) has emerged.

The metabolic programming effect was examined by screening for differences in hepatic proteome at hatch. Four proteins spots were upregulated, whereas 4 others were downregulated in the albumen-deprived chicks. To investigate the biological significance of these results, the IPA software was implemented to identify key canonical pathways. As expected, several pathways involved in the catabolism of amino acids such as valine, methionine, cysteine and glutamate were enriched in our dataset. Downregulation of mitochondrial 3-hydroxybutyrate dehydrogenase (HIBADH) and upregulation of dihydrolipoyl dehydrogenase (DLD) was detected. These enzymes are involved in the degradation of branched-chain amino acids such as valine, leucine and isoleucine. HIBADH converts 3-hydroxy-2-methylpropanoate to 2-methyl-3-oxopropanoate, whereas DLD is a component of several enzyme complexes such as the branched chain α-keto acid dehydrogenase involved in the catabolism of leucine, isoleucine and valine. Cystathionine gamma-lyase (CTH) was upregulated and is involved in the last step in the trans-sulfuration pathway from methionine to cysteine and can be both involved in the degradation of methionine and cysteine. Finally, upregulated glutamate dehydrogenase 1 (GLUD1) is involved in the biosynthesis or catabolism of glutamate and catalyzes the oxidative deamination of glutamate to 2-ketoglutarate which is an important intermediate in the TCA cycle. The observed increase in amino acids catabolism may be necessary to compensate for the lack of energy available caused by the embryonic undernutrition. As the albumen removal decreased the protein availability at the end of the incubation, changes in the expression of enzymes involved in amino acid metabolism could be foreseen. Indeed, a previous study also found indications of an altered protein metabolism in broilers treated by albumen removal before incubation, suggesting a transient increase in muscle proteolysis [8].

Metabolic programming caused by prenatal protein undernutrition was revealed by the observed hepatic proteome changes related with glucose metabolism. Upregulation of L-lactate dehydrogenase B chain (LDHB) suggests an increased conversion of pyruvate to lactate in the absence of oxygen in the liver. This lactate can subsequently be converted to glucose by the gluconeogenesis. Two of the regulatory enzymes of the latter pathway were dysregulated in the liver. Fructose-1,6-biphosphatase 1 (FBP1) was downregulated, whereas phosphoenolpyruvate 2 (PCK2) was upregulated. FBP1 catalyzes the conversion of D-fructose-1,6-biphosphate to D-fructose-6-phosphate, whereas PCK2 catalyzes the conversion of oxaloacetate to phosphoenolpyruvate, the rate-limiting step. Upregulated α-enolase (ENO1) is also involved in both the gluconeogenesis and glycolysis and catalyzes the conversion of 2-phospho-D-glycerate to phosphoenolpyruvate. In addition, glucose transporter 1 (GLUT1), a facilitative low capacity/high affinity glucose transporter across the plasma membrane was upregulated. Hepatic GLUT1 is primarily involved in cellular uptake of glucose from the plasma into the hepatocytes when nutrients are in reduced supply [20]. As no differences in plasma glucose or lactate levels were found, it is not clear whether the gluconeogenesis pathway in general is up- or downregulated.

Furthermore, a general upregulation of the TCA pathway was observed, from the upregulation of several enzymes involved such as DLD, aconitate hydratase (ACO2) and malate dehydrogenase 1 (MDH1). DLD is a component of the pyruvate dehydrogenase complex, which converts pyruvate, originating from the breakdown of carbohydrates to acetyl-CoA, the input for the TCA cycle. ACO2 catalyzes the isomerization of citrate to isocitrate via cis-aconitate, and MDH1 the conversion of malate to oxaloacetate. As previously stated, upregulation of GLUD1 leads to increased production of 2-ketoglutarate, an important intermediate of the TCA cycle. Upregulation of the TCA cycle leads to an increase in energy generation from the oxidation of acetate, derived from carbohydrates, fat and proteins.

As synthesis and degradation of **glycogen** are vital for embryonic survival during the last phase of incubation [21], glycogen levels were determined in liver, the most metabolically active tissue of the embryo. Although no significant effects of treatment or an interaction with age could be observed, the albumen-deprived chicks had lower hepatic glycogen content at hatch as compared to both the control and the sham group, suggesting that the released glucose can be distributed to extra-hepatic tissue or be used as an energy source in the liver. The variation in glycogen content between individual chicks, however, was very high, but since chicks were randomly selected for sampling, individual chicks may exhibit different hatch times and it is known that time of hatching has an impact on hepatic glycogen content [22]. Reduced liver glycogen content at hatch can be caused by either an increased use of glycogen during the hatching process or a decreased build-up of glycogen before hatch, although the latter is not likely since no differences in glycogen content were detected at ED20. It seems that the albumen-deprived chicks had to degrade more of the hepatic glycogen content, needed as energy source during the energy demanding hatching process [21].

Interestingly, several of the affected proteins are involved in glucocorticoid receptor (GR) signaling. Glucocorticoid hormones have a central role in the regulation of the glucose metabolism and bind to the GR, which is a transcription factor for regulating gene expression. In sheep, glucocorticoid receptor expression is increased in the liver of neonatal offspring born to ewes which were nutrient restricted during early-mid-gestation [23]. Offspring of rat dams that were protein-restricted throughout gestation had increased glucocorticoid receptor protein and mRNA expression in liver during fetal and postnatal life [24], [25]. The GR was not identified amongst the upregulated proteins, yet may not appear in a 2-D DIGE gel due to its membrane location. However, several proteins involved in GR signaling were upregulated. The level of plasma corticosterone, an important glucocorticoid in birds, however, was not affected in our model.

An upregulation of ENO1 and GLUT1 can be associated with increased TR/RXR activation. The thyroid hormone receptor (TR) is usually found as a heterodimer with RXR (retinoid X receptor) and regulates gene expression. Thyroid hormones are involved in a range of biological processes such as growth, development and metabolism. Plasma thyroid hormone concentrations, both T_3_ and T_4_, are reference measurements for evaluating the level of metabolism of the embryos [26]. Plasma T_3_ concentrations, the biologically active form of thyroid hormone, however, did not differ between groups, although the T_4_ concentrations at ED20 were significantly lower for the albumen-deprived embryos as compared to the control, indicating a decreased metabolism. The sham embryos had an intermediate plasma T_4_ value.

Some of the affected proteins (i.e. GLUT1, FBP1 and PCK2) have previously been associated with effects of prenatal protein undernutrition in mammalian models and will be discussed in more detail.

The GLUT proteins are a family of facilitative transport proteins, catalyzing glucose uptake across the plasma membrane, the rate-limiting step in glucose metabolism. In mammals, GLUT1 is expressed ubiquitously and facilitates the basal glucose uptake, which is essential for growth and development in most cells [27]. Expression of GLUT1 has previously been examined in other mammalian models of prenatal undernutrition, but no differences could be detected [28], [29]. Chickens exhibit a peculiar glucose transport and glucose homeostasis, since they are lacking GLUT4, the major insulin-responsive transporter [30]. The mechanism for regulation of blood glucose concentration in chickens is not well understood. Furthermore, no information is available on the roles of the chickens GLUT isoforms in relation to glucose metabolism. Most likely, GLUT1 will act in maintaining basal glucose transport in most chicken cell types as in mammals, however the precise function of GLUT1 in the chicken remains to be elucidated [27].

Chickens maintain an elevated level of blood glucose (10 mM), which is supported by high rates of gluconeogenesis. In the present study, prenatal protein undernutrition caused an increase in PCK2 protein abundance, as opposite to the decreased FBP1 protein abundance and the decreased glycogen content. The liver glycogen content has previously been shown to change in a reciprocal way to the cytosolic PCK activity [31]. Indeed, there are two forms of PCK found in most species, differing in their cellular localization: cytosolic PCK1 and mitochondrial PCK2. The relative abundance of both isoforms is dependent on the animal species and the growth stage of the animal (review [32]). In the avian liver, the mitochondrial PCK activity is the most abundant one but during the perinatal period the cytosolic PCK activity increased considerable from a few days before hatching to 4 days after hatching [31]. As PCK1 is the most abundant form in rats and has been linked previously with effects of prenatal protein undernutrition, the expression of both PCK1 and PCK2 was measured. Both genes were present in similar amounts in the liver at hatch, but were not influenced by the applied treatment.

Rats and mice have been used extensively to examine the effects of the maternal diet on the programming of the progeny. In rat dams fed a protein-restricted diet an increase in PCK1 mRNA and increased activity was detected in liver of the progeny until 11 months of age, suggesting that programming of the metabolism also extends to the regulation of gene expression [33]. In the offspring of intrauterine growth retarded rats by uteroplacental insufficiency, the hepatic expression of both PCK1 and FBP1 were increased [34]. A persistent increase in the gene expression of hepatic PCK, catalyzing the first, committed step of gluconeogenesis, leads to reduced ability of insulin to suppress hepatic glucose output, a change which is characteristic of type 2 diabetes [33]. Conversely in mink, it has been shown that feeding a low protein diet to the dam reduced the FBP1 mRNA expression in the liver of the offspring. The expression of PCK1, however, was not affected [35]. The latter is in agreement with the present study where a reduced FBP1 protein level and a trend for decreased FBP1 mRNA level was found.

Since in mammalian models of prenatal protein undernutrition, differences in gene expression are often found, the mRNA expression of the differential proteins was measured to examine if the same might be true for programming effects in the chicken. Expression of PCK2 and HIBADH, however, was not altered between different groups. The gene expression of FBP1 tended to be numerically lower (15% reduction) in the liver of the albumen-deprived chicks, compared to a 40% reduction in protein abundance. Most likely, the different abundance in these proteins are regulated via post-transcriptional or post-translational modifications.

Finally, the lower embryonic survival of the albumen-deprived chicks compared to both the sham and the control group is in agreement with studies in literature after performance of similar egg manipulations [6], [36] and with our previous results [8], [9]. The reduced survival is the result of an increased early embryonic mortality caused by the manipulation of the egg and inherent to this animal model. Furthermore, if the embryo survived the early stages of the embryonic development, the chance of a successful hatch was the same in the three treatments, as no differences in mid and late death were detected.

## Conclusion

Previous results had already demonstrated the existence of both short- and long-term effects of embryonic protein undernutrition by albumen removal in the chicken. This study demonstrates that these zootechnical and physiological differences are not yet present during the perinatal period, before the conflict between the prenatal and postnatal conditions arises. Metabolic programming was revealed by the observed hepatic proteome changes related with amino acid and glucose metabolism.
